# Biomechanical evaluation of bone screw fixation with a novel bone cement

**DOI:** 10.1186/s12938-015-0069-6

**Published:** 2015-07-30

**Authors:** Tiina Juvonen, Juha-Pekka Nuutinen, Arto P Koistinen, Heikki Kröger, Reijo Lappalainen

**Affiliations:** Department of Applied Physics, University of Eastern Finland, Kuopio Campus, Kuopio, Finland; Ozics Oy, Tampere, Finland; SIB Labs, University of Eastern Finland, Kuopio Campus, Kuopio, Finland; Department of Orthopaedics Traumatology and Handsurgery, Kuopio University Hospital, Kuopio, Finland

**Keywords:** Osteoporosis, Bone cement, Mechanical test, Screw fixation, Cement augmentation

## Abstract

**Background:**

Bone cement augmentation is commonly used to improve the fixation stability of orthopaedic implants in osteoporotic bone. The aim of this study was to evaluate the effect of novel bone cements on the stability of bone screw fixation by biomechanical testing and to compare them with a conventional Simplex^®^P bone cement and requirements of the standards.

**Methods:**

Basic biomechanical properties were compared with standard tests. Adhesion of bone cements were tested with polished, glass blasted and corundum blasted stainless steel surfaces. Screw pullout testing with/without cement was carried out using a synthetic bone model and cancellous and cortical bone screws.

**Results:**

All the tested bone cements fulfilled the requirements of the standard for biomechanical properties and improved the screw fixation stability. Even a threefold increase in shear and tensile strength was achieved with increasing surface roughness. The augmentation improved the screw pullout force compared to fixation without augmentation, 1.2–5.7 times depending on the cement and the screw type. The good biomechanical properties of novel bone cement for osteoporotic bone were confirmed by experimental testing.

**Conclusion:**

Medium viscosity of the bone cements allowed easy handling and well-controlled penetration of bone cement into osteoporotic bone. By proper parameters and procedures it is possible to achieve biomechanically stable fixation in osteoporotic bone. Based on this study, novel biostable bone cements are very potential biomaterials to enhance bone screw fixation in osteoporotic bone. Novel bone cement is easy to use without hand mixing using a dual syringe and thus makes it possibility to use it as required during the operation.

## Background

Osteoporosis is the most common bone disease, which leads to millions of fractures globally every year and is a major health and economic burden. The most common osteoporotic fractures are wrist, vertebral and hip fractures [1]. Osteoporotic fracture fixation is a surgical challenge and fixation failures are common due to bone fragility [2–5].

Cement augmentation is one of the generally used methods in orthopedic surgery to improve the fixation stability of implants in osteoporotic bone. Many studies [1, 3–12] have shown that cement augmentation improves the screw fixation strength. However, there are some problems and drawbacks in the use of conventional bone cements [7]. For example, the most problematic drawbacks of polymethylmethacrylate (PMMA) is thermal necrosis and the large mismatch in stiffness between the cement and the contiguous bone [1, 4, 5, 9, 13]. However, PMMA is one of the most common polymer bone cements used in orthopedics because long term results are good [2, 11, 14–16].

Many potential novel materials, like bone cements with calcium phosphate, have already been developed to solve the fixation problems in osteoporotic bone [2, 17–20]. However, the function of these new materials is still partly unknown or problematic. In addition, complications such as thrombosis and local cell damage are associated with calcium-based injectable bone cements [2, 7, 9]. Therefore, there is still a great demand for new, more stable fixation materials and methods to overcome these problems and to improve further the fixation strength in osteoporotic bone. These new materials and methods could also reduce the treatment cost significantly and improve the patients’ quality of life.

The first aim of this study was to test novel Ca-based bone cements and compare their basic mechanical properties (compressive elastic modulus and compressive strength) and adhesion on metal surfaces with standard surface finishes (shear and tensile strength) with those of conventional cement (Simplex^®^P). The second aim of this study was to evaluate how much the fixation stability of cancellous and cortical bone screws can be improved by bone cement augmentation in the osteoporotic bone model. The hypotheses of this study was that the novel bone cements improve screw fixation stability and that their mechanical properties are comparable to conventional Simplex^®^P bone cement and suitable for osteoporotic bone.

## Methods

Bone cements, biomechanical model, basic biomechanical tests for experimental and conventional bone cement and equipment are introduced in the following. Furthermore, details of the screw pullout testing are explained. All bone cements were tested and applied according to instructions by the manufacturers and all tests were carried out at room temperature.

### Bone cements, bone models and screws

Two experimental bone cements used in this study are biostable composites: Precursor of Comp06 (Comp06) and an alternative test compound (Altcomp) consisting of a dimethylacrylate-based polymer matrix, ceramic fillers containing silicate glass and hydroxyapatite (HA) (Ozics Ltd, Tampere, Finland). These bone cements are designed for minimal invasive fracture treatment of vertebral compression fractures and to enhance the fixation of screws or other metallic osteosynthesis devices in bone with a low mineral density or osteoporosis. They are supplied in a compact and prefilled dual injectable syringe allowing accurate and fast application of uncured bone cement to the bone site. As a reference material a commercial, widely used bone cement was used; Simplex^®^P (Stryker Howmedica Osteonics by Stryker Orthopaedics, Limerick, Ireland) which is mostly used in prosthetic surgery.

**Fig. 1:**
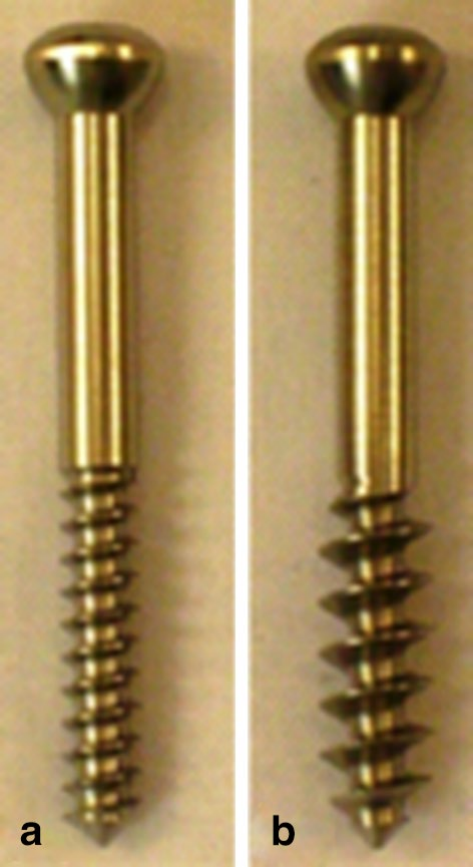
The tested bone screws: cortical (**a**) and cancellous (**b**) bone screws. The inner and outer diameters of the screws are for cortical (3.0/4.5 mm inner/outer diameter × 45 mm length) and for cancellous (5.0/6.5 mm inner/outer diameter × 45 mm length).

Osteoporotic bone model used in this study consists of porous polyurethane (PU) foam blocks (Sawbones Europe AB, Malmö, Sweden), sized 40 mm × 130 mm × 180 mm, as a cancellous bone and a 2 mm thick polycarbonate (PC) plate (Lexan Margard, GE Plastics, New York, USA) as a cortical layer. PU foam for cancellous part was cellular rigid polyurethane foam (# 1522-10) with a density of 0.16 g/cm^3^. The effect of bone cement fixation was studied for cortical (3.0/4.5 mm inner/outer diameter × 45 mm length) and cancellous bone screws (5.0/6.5 mm inner/outer diameter × 45 mm length) (Fig. 1). All screws were manufactured from stainless steel (AISI303) for test purposes (Mectalent Ltd, Oulu, Finland).

### Compressive strength and compressive elastic modulus of the bone cements

The compressive strengths of the bone cements were evaluated following the ISO 5833:2002 standard [21]. Cylinders of (6 ± 0.2) mm in diameter and (12 ± 0.2) mm in height were prepared from bone cements. The cylinders were kept (24 ± 2) hours at room temperature prior to testing and then tested in compression on a dynamic testing machine (Instron 8874; Instron Co, Canton, MA, USA) using a cross-head speed of 20 mm/min. The force applied to cause fracture, or the 2% offset load, or the upper yield-point load (highest load, where the yielding began), whichever occurred first, was recorded for each cylinder [21]. The average compressive strength of at least five cylinders was calculated. The compressive elastic modulus of each cylinder was defined as the slope of the stress–strain curve in the linear, elastic deformation region, typically calculated using the values in the range 10–90% of the yield stress. Results are presented as mean ± standard deviation.

### Adhesion testing

The adhesion of bone cement to a stainless steel surface was verified by determining the interfacial tensile and shear strength. Shear and tensile strength of metal–bone cement interfaces were measured for three different roughness: mechanically polished (*R*_a_ = 0.42 ± 0.12 µm), glass blasted (*R*_a_ = 0.78 ± 0.10 µm) or corundum blasted (*R*_a_ = 1.2 ± 0.2 µm) stainless steel surfaces. The roughness profiles were measured using a Mitutoyo SJ301 roughness tester (Mitutoyo Corp., Kawasaki, Japan).

*The interfacial shear strength* was determined according to the procedure described by Stone et al. [22]. The bone cement was injected into a mould and around a stainless steel rod (diameter 10 mm). Thus, a cement mantle of 10 mm in height and 21 mm in diameter was produced around the stainless steel rod. The test rod was pushed axially through the cylindrical cement mantle. The maximum load was recorded and transformed into interfacial shear strength since that way the results are less dependent on the size of tests specimens and the results from different studies can be compared easier by giving strength values.

*The interfacial tensile strength* was determined according to the procedure described by Keller et al. [23]. The bone cement was allowed to set between the ends of the stainless steel test rods (diameter 10 mm) inside an axially split mould. Thickness of the cement between stainless steel rod ends was 2–4 mm. The rods were either mechanically polished, glass blasted or corundum blasted to create surfaces with different roughness. The rods were separated (detached) using test machine (Instron 8874; Instron Co, Canton, MA, USA) with axial pulling. The maximum load was recorded and transformed into tensile strength. The cross-head speed of the material testing machine was in both cases 0.5 mm/min.

### Pullout testing

The pullout strength of cortical and cancellous screws was evaluated with bone cements (Comp06, Altcomp and Simplex^®^P) and without bone cement. All the pullout tests were carried out as described in our previous article [24] and based on ASTM F543-07 Standard Specification and Test Methods for Metallic Medical Bone Screws [25]. The tests were performed using a dynamic testing machine (Instron 8874; Instron Co, Canton, MA, USA). Part of the pullout tests were done by varying cement hardening time after cement injection. All pilot holes, gliding holes and tapping were carried out based on the recommended sizes for each screw type and size (Table 1). The cross head speed of the material testing machine was in all cases 10 mm/min. The synthetic bone blocks were incubated 3 days in water at 37°C prior to the testing to study the effect of moisture which is easy affecting in real operations, too. Pullout tests were performed for each screw type without/with bone cement. Each screw was pulled out along the screw axis perpendicular to the top surface of the test block until the bonding failed or the screw was released from the test block and maximum pullout force of the screw was recorded. The effect of moisture on augmented fixation was studied for Comp06 and Simplex^®^P cements with cancellous screws by presoaking bone models 24 h in distilled water, drilling the pilot hole and then immediately cementing and inserting the screw. Before pullout testing the bone blocks with the hardened cement and the screws were stored (24 ± 2) hours in distilled water at 37°C.

**Table 1:** Pilot hole diameters for cortical and cancellous bone screws without cement and with bone cements

Screw	Pilot hole diameter in PC sheet (mm)	Pilot hole diameter in block before/after cementing (mm)	Tapped
Cortical	3.0 (series 1, 3) 5.0 (series 2)	3.0/– (series 1) 5.0/3.0 (series 2) 3.0/– (series 3)	– Yes –
Cancellous	6.5^a^ (all series)	3.0/– (series 1) 5.0/5.0 (series 2) 3.0/– (series 3)	– – –

Series 1 and 2 with cement:

*Series 1* screws inserted immediately after cementing, *Series 2* screws inserted after 10 min cementing, *Series 3* without cement.

^a^For cancellous bone screw this is called gliding hole.

### Statistical analysis

Statistical comparisons were carried out using IBM SPSS Statistics software (version 19; SPSS Inc., USA). The Kruskal–Wallis test was used to compare the differences between the cements and the Mann–Whitney U test was used to evaluate the effect of bone moisturizing on the pullout strengths. *P*-values of less than 0.05 were considered statistically significant.

## Results

### Compressive strength and compressive elastic modulus

Compressive strengths of bone cements, determined according to ISO5833 standard (ISO5833 2002), are listed in Table 2. Both novel bone cements and conventional Simplex^®^P fulfilled the requirements of the standard for compressive strength (≥70 MPa). Both the compressive strength and the compressive elastic modulus of Comp06 and Altcomp were significantly higher than those of Simplex^®^P. Differences in the values for different bone cements are statistically significant (*p* < 0.05).

**Table 2:** Compressive elastic modulus and compressive strength values for all tested bone cements

Bone cement	Compressive elastic modulus ± std (GPa)	Compressive strength ± std (MPa)
Simplex^®^P	2.22 ± 0.10	78 ± 6^a^
Comp06	4.9 ± 0.3	150 ± 20
Altcomp	3.5 ± 0.6	220 ± 80

^a^2% offset yield strength

### Adhesion

**Fig. 2:**
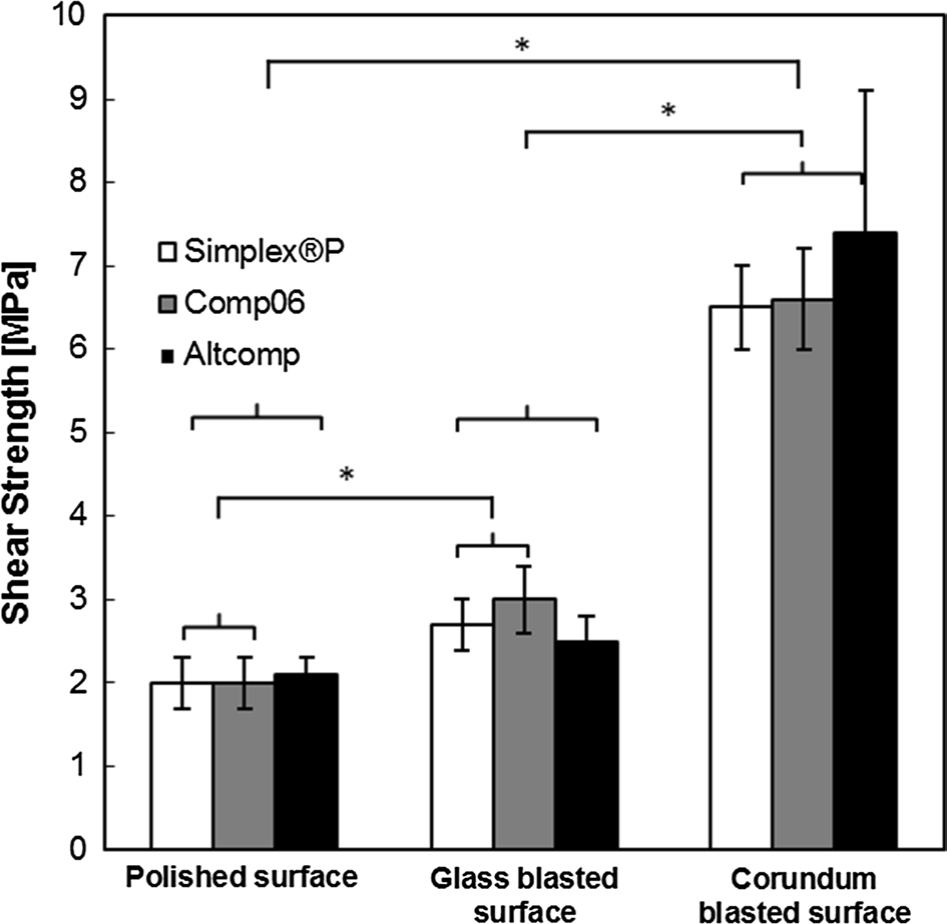
Comparison of the maximum shear strengths. The adhesion of bone cement to a stainless steel surface was verified by determining the shear strength of metal–bone cement interfaces. The maximum shear strengths were measured for three different roughness: mechanically polished (*R*_a_ = 0.42 ± 0.12 µm), glass blasted (*R*_a_ = 0.78 ± 0.10 µm) or corundum blasted (*R*_a_ = 1.2 ± 0.2 µm) stainless steel surfaces. Mean values ± standard deviation of the measured values are presented (*N* = 5) (Kruskal–Wallis, *p < 0.05).

**Fig. 3:**
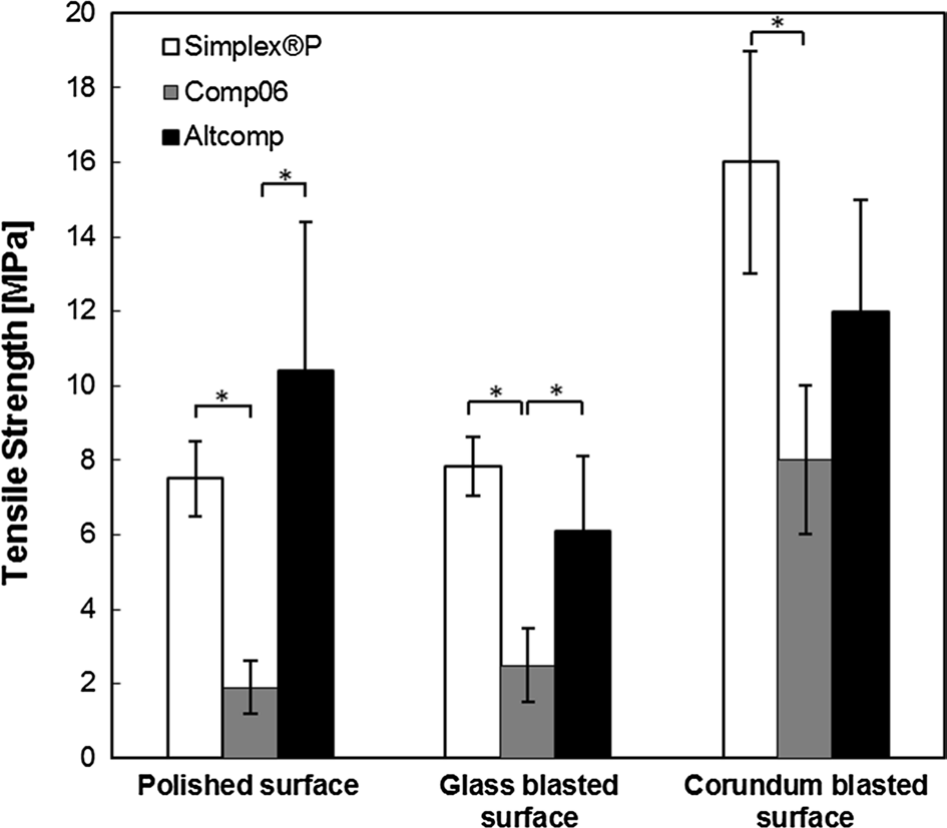
Comparison of the maximum tensile strengths. The adhesion of bone cement to a stainless steel surface was verified by determining the tensile strength of metal–bone cement interfaces. The maximum tensile strengths were measured for three different roughness: mechanically polished (*R*_a_ = 0.42 ± 0.12 µm), glass blasted (*R*_a_ = 0.78 ± 0.10 µm) or corundum blasted (*R*_a_ = 1.2 ± 0.2 µm) stainless steel surfaces. Mean values ± standard deviation of the measured values are presented (*N* = 5) (Kruskal–Wallis, *p < 0.05).

Interfacial shear and tensile strengths were measured to investigate the bone cement adhesion to polished, glass blasted and corundum blasted stainless steel surfaces. The interfacial shear and tensile strengths for all tested cements (Comp06, Altcomp and Simplex^®^P) are compared in Figs. 2 and 3. In general, the effect of surface treatment is significant (*p* < 0.05) as shown in the Fig. 2. In all the cases, the adhesive failure occurred clearly at the stainless steel metal–bone cement interface and the rest of the cement mantle remained intact.

Differences in interfacial shear strengths between the novel cements and Simplex^®^P were not significant. In contrast, the tensile strength of Comp06 was significantly lower (*p* < 0.05) than that of Simplex^®^P with all three surface roughnesses. Difference between Comp06 and Altcomp was significant (*p* < 0.05) only for polished and glass blasted surface. For example in the case of glass blasted surface, tensile strength was (2.5 ± 1.0 MPa) for Comp06, (6.1 ± 2.0 MPa) for Altcomp and (7.8 ± 0.8 MPa) for Simplex^®^P.

### Pullout strength

**Fig. 4:**
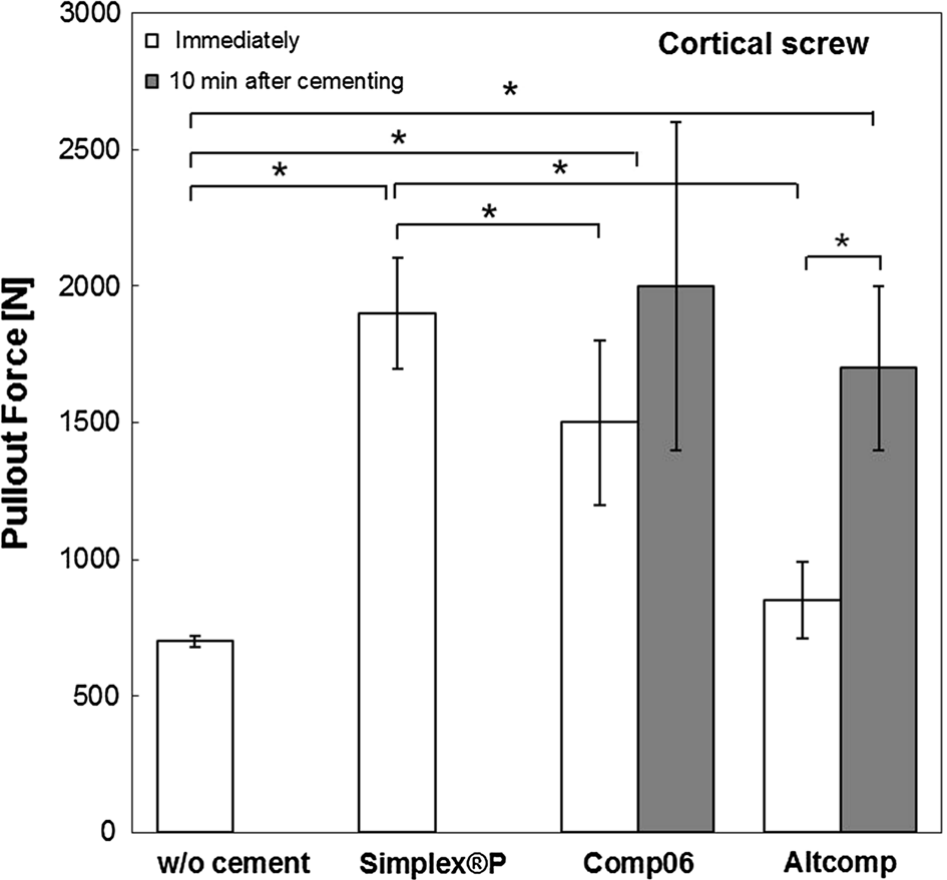
Comparison of the maximum pullout strengths of cortical screws. The pullout strength of cortical screw was evaluated with bone cements (Comp06, Altcomp and Simplex^®^P) and without bone cement. Mean values ± standard deviation of the measured values are presented (*N* = 5) (Kruskal–Wallis, *p < 0.05).

**Fig. 5:**
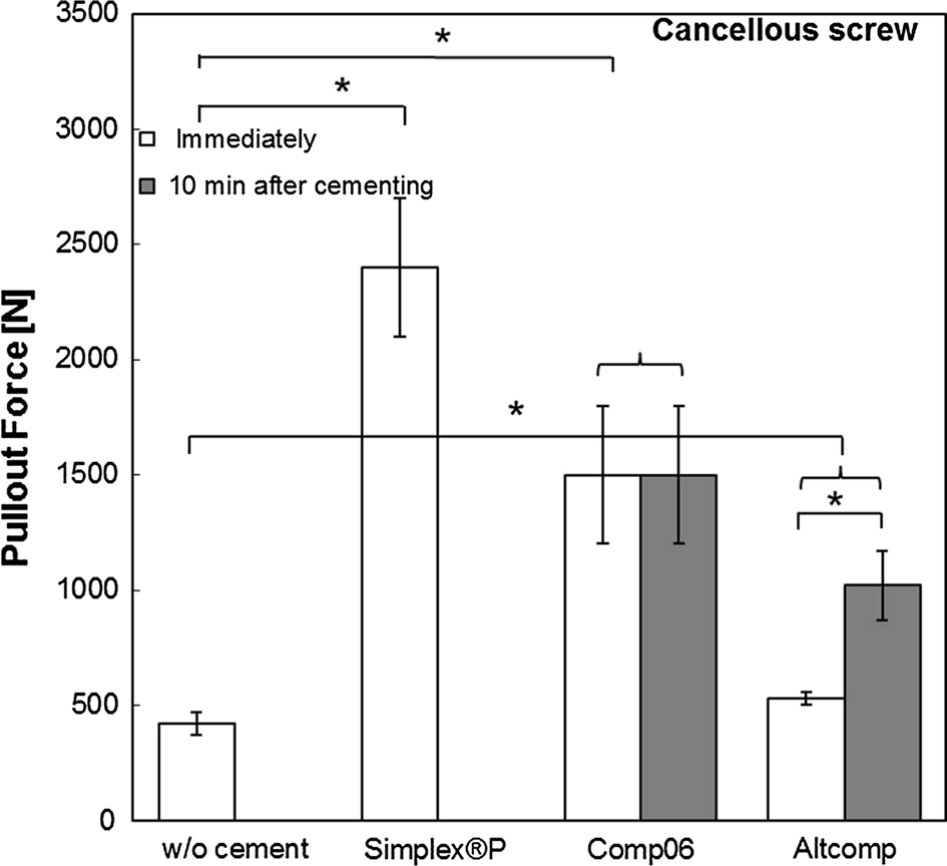
Comparison of the maximum pullout strengths of cancellous screw. The pullout strength of cancellous screw was evaluated with bone cements (Comp06, Altcomp and Simplex^®^P) and without bone cement. Mean values ± standard deviation of the measured values are presented (*N* = 5) (Kruskal–Wallis, *p < 0.05).

The effect of bone cement augmentation on screw fixation stability was evaluated with the pullout testing. The bone cement augmentation improved significantly the screw pullout force with all the tested bone cements and screw types (*p* < 0.05), Figs. 4 and 5. The average pullout force for cortical screw with Altcomp bone cement increased by a factor of 2 as the screw was inserted in tapped hole of hardened cement. Similar trend was observed with Altcomp with cancellous screws (without tapping). In the case of Comp06, time between cementing and screw insertion did not improve pullout force significantly.

The effect of moisture on screw fixation was studied for cancellous screw with Comp06 and Simplex^®^P bone cements. Results (mean ± standard deviation) are presented in Table 3. Reference values for dry conditions are also included. The improvements in pullout forces for these cements were comparable with the improvements without moisturizing. Significant differences were not found between cements or moist and dry conditions (Mann–Whitney U).

**Table 3:** The effect of moisture on pullout forces for cancellous screw with Comp06 and Simplex^®^P cements

Cement	Pullout force ± std (N) Moist	Pullout force ± std (N) Dry
Comp06	1,600 ± 500	1,500 ± 300
Simplex^®^P	1,800 ± 600	2,500 ± 300

## Discussion

Bone cement augmentation is an effective and common method to increase the fracture fixation stability, especially in osteoporotic bone [1, 3–11]. Nevertheless, there are still some problems related to conventional bone cements, such as thermal necrosis of tissues and poor long term biocompatibility. New and better materials are needed to improve the fixation strength and the applicability of bone cements for osteoporotic bone. For example, the optimal fixation method and bone cement for osteoporotic bone and for normal healthy bone are not the same [26].

This study was carried out to evaluate the effect of novel biostable bone cement augmentation on the fixation stability in the osteoporotic bone model by testing and comparing the biomechanical properties of the biostable bone cements with those of conventional Simplex^®^P bone cement. Based on the testing, the cement augmentation improved the maximum pullout force 1.2–5.7 fold depending on the cement and on the screw type. The results were reproducible and the improved fixation stability is expected in clinical practices, too. The improvements achieved here are comparable to those found in the literature typically by a factor of 1–5 [3, 4, 7, 10, 11] and in our earlier study [24] by a factor of 3–12 increase in pullout force. However, the comparison to other results is not straightforward because the improvements achieved by bone cement augmentation depend on several factors, such as test parameters, bone model and screw type [4, 10, 26–28].

This study showed that these novel biostable bone cements are potential alternatives for conventional bone cement. The advantages of these novel bone cements include: simple handling properties, injectability, low polymerization temperature and good biomechanical properties, such as compressive strength and optimal working and setting time. They are easy to use without hand mixing and can be delivered in a controlled manner by a prefilled dual injectable syringe. Both tested experimental bone cements fulfilled the requirement of the ISO 5833 standard [21] for compressive strength (≥70 MPa). The measured value of compressive strength for Simplex^®^P (78 ± 6 MPa) fulfilled also the requirement of the standard and was in good agreement with the literature [13, 20, 29]. The biomechanical properties, especially the compressive strength (150 ± 20 and 220 ± 80 MPa, for Comp06 and Altcomp, respectively) and compressive elastic modulus of these novel bone cements (4.9 ± 0.3 and 3.5 ± 0.6 GPa, respectively) exceeded those of conventional Simplex^®^P (78 ± 6 MPa and 2.2 ± 0.1 GPa) and of other conventional bone cements based on literature values [13, 20, 29, 30]. These experimental bone cements are designed for augmented screw fixation in osteoporotic bone. Since their biomechanical properties are between those of cancellous and cortical bone, in principle they could be used with both cortical and cancellous screws. Furthermore, hydroxyapatite and good flow properties help to achieve maximum interdigitation with the trabecular bone. The biomechanical properties of bones vary depending on e.g. the skeletal site, on the bone mineral density and on the testing method. Cancellous bone material properties are inhomogeneous and anisotropic. For example, the compressive strength of cancellous bone varies from 0.1 to 30 MPa [31, 32] and elastic modulus from 0.1 to 3.5 GPa [31, 32]. Correspondingly for cortical bone the compressive strength varies from 131 to 205 MPa (strength) [33] and from 10 to 18 GPa (modulus) depending on the loading direction [31, 33].

This study showed that initial screw fixation stability with the novel biostable bone cements was comparable with that of the conventional Simplex^®^P bone cement. However, this requires hardening of the novel cements and tapping of the pilot hole. The use of a tapping tool reduces the risk to damage fragile osteoporotic bone. Interestingly, the increase in pullout force in the case of cancellous screws was higher for Simplex^®^P than for novel bone cements, but the improvements achieved by the cements were about the same for cortical screws. Furthermore, the moisture in the bone model material did not affect the fixation strength significantly. Functional fixation withstands loading forces and restores initial strength of bone but at the same time it should enable new bone formation. The optimal stiffness and strength of the bone cement are still unclear and more research is needed to overcome the problems like difficult handling and poor biocompatibility of conventional bone cements in osteoporotic bone [4, 11, 13]. In principle, composite approach of novel bone cements allows to fine tune their biomechanical properties for each fixation type and local bone properties. Polycabrolactone (PCL) bone cement used in our earlier study [24] had significantly different biomechanical properties, i.e. it was much softer (compressive strength: 27.7 ± 1.2 MPa) and less stiff (compressive elastic modulus: 480 ± 20 MPa) than bone cements tested in this study. In spite of this, the results of this study were comparable to those with PCL, especially when taking into account the less dense (0.12 g/cm^3^) bone model used for PCL. Pullout force of cancellous screw in cellular cell bone model with and without PCL was (1510 ± 230) N and (180 ± 100), respectively [24]. It seems that bone cement augmentation is beneficial for initial stability although the cement and its biomechanical properties vary.

Surface roughness and the viscosity, the shrinking and the porosity of the cement affect adhesion at the metal–cement interface [34–38]. Thus, the bone cement viscosity was indirectly compared by determining the adhesion strength in this study. Variations in interfacial tensile strength values between bone cements were higher than in interfacial shear strength. Interfacial tensile strength turned out to be more sensitive to the properties of surface and to the behaviour of the cement. In general, cement–bone interface is stronger in shear than in tensile loading [38] as found in this study, too. Higher metal surface roughness increases the effective surface area and allows easier removal of air from the cement–metal interface leading to better adhesion and more reproducible results as Müller et al. [34] found, too. Lower viscosity improves cement infiltration into the bone and improves the fixation [9, 35–37]. Müller et al. [34] have shown also that the high viscosity cement does not infiltrate sufficiently into the surface roughness and trapped air decreases the effective contact area between metal and cement [34] which is in agreement with our findings. The use of highly viscous cement requires large injection pressure to ensure good penetration into the bone. On the other hand, increasing the cement viscosity and using the low flow rate decrease the risk of extravasation. Extravasation depends on the bone structure (e.g. porosity, the bone permeability, bone pore size) but also on the cement viscosity [39]. Cement is flowing out of the treatment area into the surrounding tissues which may lead to unstable fixation, tissue reactions etc. In the case of the novel biostable bone cements of this study, the viscosity of bone cement was adjusted to reach a compromise, i.e. low enough to allow relatively easy manual injection but high enough to prevent extravasation. This resulted in good control during the injection phase and low pressure injection which are both beneficial properties.

There were some limitations in this study. One obvious limitation was the use of synthetic bone model instead of real bone. The low density PU foam model was chosen for this study because of many advantages, such as homogeneity in mechanical properties and ease of use compared to real human or animal bone [22]. The properties of low density PU foams are proved to be suitable especially to model osteoporotic bone [28, 40]. Cancellous bone density varies across individuals and depends significantly from anatomic site from 0.09 to 1.26 g/cm^3^ [40]. Thus, PU foam with the density of 0.16 g/cm^3^ used in this study models highly stage osteoporotic cancellous bone.

One limitation of this study was the relatively small number of adjacent samples (N = 5) due to practical limitations. However, the expected and observed differences between the different sample sets were so large that the statistical power was typically well above 80%.

The cortical layer is one critical factor both in modeling and in clinical case because it can significantly increase pullout force by preventing pullout of a cement mantle supporting the screw [41]. Procter et al. [41] have studied the effect of the cortex on the pullout strength. They observed that with the cortical layer the increase in pullout force was nearly 14-fold but without cortical layer the increase was only fourfold [41]. In this study, we used 2 mm thick polycarbonate layer to model cortical bone and found that a sufficient cement plug provides “a stable anchoring mantle” in the porous PU foam and cortical layer increases the pullout force. The depth of penetration and the amount of cement are also critical factors [9, 11, 37, 42]. Stadelmann et al. [7] have shown that the more fragile bone is, the deeper penetration is needed to achieve stable enough fixation. The penetration of the bone cement inside the structure of cellular rigid PU foam (also used in our earlier study [24]) corresponds to the real clinical situation better than the penetration inside the open cell foams [28]. Additionally, the pullout forces without cement had a smaller standard deviation than with bone cement. This is probably due to more affecting factors with cement such as cement–bone block interface and variation in infiltration.

Cement hardening before screw insertion can significantly affect fixation stability. In our study, the cement hardening increased the pullout force for both novel bone cements with cortical screw. In some studies [11, 15, 43], the effect of cement hardening on pullout force have been evaluated. For example, Flahiff et al. [11] have shown that screws placed in doughy cement had a significantly higher pullout force than those placed in soft or hard cements. In our case, screw hole in hardened cement was drilled and tapped for cortical screws, which seems to be beneficial for fixation and allowed easy insertion of screws.

In the future, one relevant topic would be to study long-term effects of the bone cement, e.g. fatigue behavior in clinical use. However, initial clinical results have been promising. Surgeons who have used this novel bone cement, Comp06, have reported that it is easy to use, interdigitates well with cancellous bone and provides pain relief without any observed adverse events (Personal communication, Dr Razzano M, Colleferro, Rome, Italy, December 21, 2012). Proper viscosity of the material helps to avoid extravasation or venous leakage. Low exothermic reaction during setting, high strength, stability of the implant thus allowing early controlled mobilization of the patient and the ready to use on demand are advantages of these types of novel bone cements (Personal communication, Dr. Ricciardi A, Castelfranco Veneto, Italy, December 12, 2012).

## Conclusion

The aim of this study was to show whether the bioactive bone cement could improve the screw fixation strength in osteoporotic bone model. Novel biostable bone cement (Comp06) provided significantly improved fixation of screws in the osteoporotic bone model based on screw pullout testing. Based on this study, fixation stability in osteoporotic bone is difficult to model properly since bone cement, screw type and test materials etc. affect the fixation stability. Finally, the improvements achieved with novel bone cements demonstrated their potential to increase the fixation stability in osteoporotic bone compared to conventional bone cement Simplex^®^P. Good (bio)mechanical properties exceed the requirements of industry standards. The unique mechanical properties combined with HA-content make these novel bone cements more biological than PMMA bone cement.
